# Effect of Off-Season Flooding on Growth, Photosynthesis, Carbohydrate Partitioning, and Nutrient Uptake in *Distylium chinense*

**DOI:** 10.1371/journal.pone.0107636

**Published:** 2014-09-15

**Authors:** Zebin Liu, Ruimei Cheng, Wenfa Xiao, Quanshui Guo, Na Wang

**Affiliations:** State Forestry Administration Key Laboratory of Forest Ecology and Environment, Research Institute of Forest Ecology, Environment and Protection, Chinese Academy of Forestry, Beijing, PR China; University of Nottingham, United Kingdom

## Abstract

*Distylium chinense* is an evergreen shrub used for the vegetation recovery of floodplain and riparian areas in Three Gorges Reservoir Region. To clarify the morphological and physiological responses and tolerance of *Distylium chinense* to off-season flooding, a simulation flooding experiment was conducted during autumn and winter. Results indicated that the survival rate of seedlings was 100%, and that plant height and stem diameter were not significantly affected by flooding. Adventitious roots and hypertrophic lenticels were observed in flooded seedlings after 30 days of flooding. Flooding significantly reduced the plant biomass of roots, net photosynthetic rate (*P*_n_), stomatal conductance (*g*_s_), transpiration rate (*T*_r_), maximum photochemical efficiency (Fv/Fm), photochemical quenching (qP), and electron transport rate (ETR) in leaves, and also affected the allocation and transport of carbohydrate and nutrients. However, *D. chinense* was able to maintain stable levels of *P*_n_, Fv/Fm, qP, ETR, and nutrient content (N and P) in leaves and to store a certain amount of carbohydrate in roots over prolonged durations of flooding. Based on these results, we conclude that there is a high flooding tolerance in *D. chinense*, and the high survival rate of *D. chinense* may be attributable to a combination of morphological and physiological responses to flooding.

## Introduction

Flooding is a common environmental stress; natural phenomena such as rainfall, snowmelt, or tides and human activities such as the construction of tidal water conservancy and hydropower can result in flood-prone environments [1], [2]. Flooding stress alters the original growth environment and conditions experienced by plants, greatly affecting plant growth and physiological rhythms. The rapid reduction in oxygen available for roots resulting from flooding is a major factor restricting plant survival, growth and development [3]. There are many example for effects of flooding such as the inhibition of the growth of roots, shoots, and new leaves, in turn causing decreased growth in the entire plant; reductions in the net photosynthetic rate, photosynthetic electron transport rate, and photosystem II (PSII) photochemical efficiency [4]; reactive oxygen species (ROS) metabolism disorders [5]; reductions in element uptake; and inhibition of transport from roots to leaves [6]. However, some plant species can sense low oxygen levels via the posttranslational regulation of key hypoxia-responsive transcription factors by the N-end rule pathway to enhance plant responses to hypoxia and flooding [7], [8], [9] and bring about various morphological, physiological, and biochemical changes that improve flood tolerance [10],[11]. These changes include alteration of the light absorption efficiency of plants through changes in leaf morphology with a reduction of light intensity [12], the formation of adventitious roots and hypertrophic lenticels at the stem base [13], the reopening of stomata [14], the formation of a complex antioxidant defense system [15], and reductions in carbohydrate consumption and changes in nutrient partitioning [16].

The construction of the Three Gorges Dam reservoir spans the Yangtze River, China, forming the hydro-fluctuation belt with a drop in water level of nearly 30 m and an area of 300 km^2^. The water level of the hydro-fluctuation belt would fluctuate from 145 m in summer to 175 m in winter. This hydrologic regime is the opposite of the natural hydrologic regime of Yangtze River before the Three Gorges Dam construction when the peak flows occurred in summer and low flows occurred in winter [17]. With the rise of the water level in autumn, plants living within the belt would face a complex flooding environment, and some flood-intolerant plants gradually died off, exacerbating vegetation habitat fragmentation and the degradation of the water-fluctuation zone, and deleteriously affecting biological diversity, ecosystem structure, and reservoir function [18]. Therefore, a pressing scientific challenge is to recover and restore the vegetation there and ensure proper ecological function [19]. Previous studies have shown that native trees play an important role in maintaining the ecological environments of reservoir areas and in ensuring normal ecosystem function [20]. Thus, screening for native species offers a quick and effective approach to the recovery of the ecological environment of the hydro-fluctuation belt.

*Distylium chinense* (Fr.) Diels is an evergreen shrub belonging to the Hamamelidaceae family. This species is ideal for solid earth embankments due to its strong root system, erosion tolerance, flooding tolerance, and resistance to sand burial soaks [21]. Chen et al. [22] demonstrated that *D. chinense* has a strong tolerance to flooding, with the ability to withstand up to 6 months of inundation. However, little is known about the morphological and physiological responses of *D. chinense* to off-season flooding. The mechanisms driving the response of *D. chinense* to autumn and winter flooding will help to understand how this species can grow and survive and to provide a theoretical basis for the vegetation screening and recovery of the hydro-fluctuation belt. Meanwhile, this information can help elucidate the adaptive strategies of plants to off-season flooding. Therefore, the main objectives of the present study were to assess the morphological and physiological changes to off-season flooding and to investigate the relationships between these changes and the tolerance of *D. chinense* to flooding.

## Materials and Methods

### Plant materials and experimental design

*D. chinense* is perennial shrub, belonging to the genus *Distylium* of the Hamamelidaceae. Young branches stout, internodes 2–4 mm, older growth glabrescent. Petiole 1.5–2 mm, densely lepidote; leaf blade elliptic to oblanceolate, 2–4 cm long and 1–1.2 cm wide, both surfaces glabrous, base broadly cuneate, margin entire or with 2 or 3 teeth on each side near apex, apex subacute; lateral veins 5 on each side, reticulate veins obscure on both surfaces. It begins growth in late autumn; its shoots begin to flower in early spring, and then produce seeds in autumn [23].

On 1 August 2012, 2-year-old *D. chinense* plants were transplanted into plastic pots (22 cm wide ×17 cm height) with 3.5 kg of local loam. All seedlings were planted at the Forest Ecology and Environment Research Station of the Chinese Academy of Forestry, Yichang, Hubei Province, China (30°53′ N, 110°54′ E, 296 m a.s.l.). All plants were grown under the same conditions. On 1 October 2012, after a 60-day acclimation period under well watered and well drained routine conditions, an outdoor experiment was conducted, 100 seedlings of uniform size (46.6±0.83 cm in height, 6.08±0.17 mm in diameter) were selected and randomly assigned to two groups of 50 seedlings. One group was flooded to about 5 cm above the soil surface, and the other group was used as non-flooded controls, which was watered and well drained. Flooded plants were placed in a pool divided into five small pools(4 m long×0.75 m wide×0. 4 m high)for the flooding treatment, ten plants were placed in each small pool. During the experiment, air temperature and relative humidity ranged from 5°C to 27°C and 54% to 82%, respectively. On days 7, 15, 30, 45, and 60 after initial flooding, five plants from randomly selected pots(five flooded plants came from different pools, the same below)were harvested. Harvested samples were separated into leaves, stem, and roots and dried at 70°C for 48 h. After biomass was determined, leaves and roots were ground for further nutrient analysis. At the end of flooding, three plants per treatment were harvested and separated into leaves, stems, and roots for nonstructural carbohydrate analysis. Plant material was then dried at 105°C for 15 min, followed by 48 h at 70°C.

### Morphological characteristics

Adventitious roots and lenticels of stems were observed on days 7, 15, 30, 45, and 60 after initial flooding. Nine seedlings were randomly selected (two flooded seedlings were randomly selected from each pool; the branches of three flooded seedlings in a pool were inadvertently crushed at the last sampling, so only one seedling from this pool was used to study the morphological characteristics of the plants) to record the number of adventitious roots and lenticels of stems, and measure plant height and stem diameter using measuring tape and digital vernier caliper, respectively, at the end of flooding.

### Photosynthesis

On days 7, 15, 30, 45, and 60 after initial flooding, the third or fourth upper leaf from each seedling crown was chosen for measuring the photosynthesis parameters of net photosynthetic rate (*P*_n_), stomatal conductance (*g*_s_), transpiration (*T*_r_), ambient CO_2_ concentration (*C*_a_), and intercellular CO_2_ concentration (*C*_i_) using a Li-6400XT portable photosynthesis system (Li-Cor, Inc., Lincoln, NE, USA). Measurements were conducted between 09:00 and 11:30 h. The saturated light intensity was set at 1000 µmol·m^−2^·s^−1^. The temperature of the leaf chamber was maintained at about 20°C, and CO_2_ concentration was maintained at about 400–410 µmol·mol^−1^. The stomatal limiting value was modeled as *L*_s_  = 1– *C*_i_/*C*_a_ [24]. Three leaves from each of three plants per treatment were randomly selected for measurements. The area of the tested leaf was traced on a piece of paper and then the area of the paper was determined with Li-3000 leaf area meter (Li-Cor, Inc., Lincoln, NE, USA).

### Chlorophyll fluorescence parameters

Chlorophyll (Chl) fluorescence parameters were measured using the fluorescence leaf chamber of a Li-6400XT portable photosynthesis system on days 7, 15, 30, 45, and 60 after initial flooding. The fluorescence parameters measured were maximum photochemical efficiency (Fv/Fm), photochemical quenching (qP), non-photochemical quenching (qN), and electron transport rate (ETR). Three leaves from each of three plants per treatment were randomly selected for measurements, and all sampled plants were dark-adapted for at least 1 h prior to measurements.

### Nonstructural carbohydrates

Nonstructural carbohydrates were measured using a Waters 2695 High-Performance Liquid Chromatography system (Waters, Inc., Milford, MA, USA) under the following chromatographic conditions: chromatographic column: Sugar-Pak 1 (Waters, Inc.); mobile phase: water; flow: 0.6 ml·min^−1^; column temperature: 70°C; detector: parallax detector. For the extraction of soluble sugar [25], 0.5 g of dried plant material was weighed and then digested for 2 h with 50 ml of distilled water. Samples were brought up to a constant volume and then filtered. For the extraction of starch [25], 0.5 g of dried plant material was weighed and refluxed for 8 h in a 100-ml water bath of distilled water and 10 ml 2∶1 (v:v) hydrochloric acid. The samples were then neutralized with 40% sodium hydroxide, brought up to a constant volume, and filtered.

### Nutrient analyses

Plant samples were oven dried at 70°C to a constant weight. Nitrogen (N) was analyzed using a UK152 Distillation & Titration Carlo Erba N analyzer (Velp Scientifica, Usmate (MB), Italy). Phosphorus (P), iron (Fe), and manganese (Mn) were determined using an IRIS IntrepidII XSP Plasma Emission Spectrometer (Thermo Fisher Scientific, Waltham, MA, USA), and the tissues were digested by adding 10 ml of nitric acid prior to analysis.

### Data analysis

All statistical analyses were performed using SPSS 19.0 software (SPSS Inc., Chicago, IL, USA). A t-test was used when experimental treatments were compared as described by Pociecha et al. [26].

## Results

### Morphology and growth

Throughout a period of 60 days of flooding, the survival rates of seedlings were 100% with the exception of slight chlorosis of older leaves and reduced generation of new leaves. The formation of adventitious roots and hypertrophied lenticels was observed in flooded seedlings after 30 days of flooding, and the number of adventitious roots and hypertrophied lenticels was 6.10 and 33.2, respectively, by day 60 (Table 1).

**Table 1:** Morphological characteristics of *D.chinense* seedlings after 60 days of flooding (means ± S.E., n = 5).

Treatments	Plant height(cm)	Stem diameter(mm)	Number of adventitious roots	Number of hypertrophied lenticels
Non-flooded	48.14±0.50	6.67±0.14	-	-
Flooded	48.47±0.86 NS	7.06±0.23 NS	6.10±1.28	33.2±6.46

Flooding had no effect on plant height or stem diameter (Table 1). The heights of non-flooded and flooded seedlings were 48.14 and 48.47 cm, and stem diameters were 6.67 and 7.06 mm, respectively, by the end of the experiment. No significant reductions in stem and leaf biomass of flooded seedlings were observed within 60 days of flooding, whereas significant (P<0.01) reductions in root biomass occurred after 30 days of flooding, with a 40.2% reduction compared to non-flooded seedlings at the end of the flooding experiment. Consequently, total biomass and the root mass ratio of flooded seedlings were significantly (P<0.05) lower than in non-flooded seedlings after 30 days of flooding (Table 2).

**Table 2:** Biomass characteristics (means ± S.E., n = 5) of *D.chinense* seedlings under non-flooded and flooded conditions.

Characteristics	Treatment	Time (days)				
		7	15	30	45	60
Root biomass (g)	Non-flooded	6.01±0.16	6.62±0.45	7.15±0.36	8.66±0.43	9.15±0.55
	Flooded	6.07±0.27 NS	5.53±0.24 NS	5.21±0.22**	4.94±0.39**	5.74±0.21**
Stem biomass (g)	Non-flooded	4.89±0.23	5.43±0.33	5.64±0.41	6.05±0.48	6.22±0.26
	Flooded	4.77±0.15 NS	4.85±0.34 NS	5.55±0.36 NS	5.90±0.49 NS	6.15±0.27 NS
Leaf biomass (g)	Non-flooded	4.43±0.09	5.71±0.39	5.80±0.40	5.84±0.27	6.94±0.42
	Flooded	4.68±0.10 NS	5.31±0.18 NS	5.24±0.31 NS	5.59±0.37 NS	5.61±0.37 NS
Total biomass (g)	Non-flooded	15.32±0.44	17.76±0.99	18.59±1.05	20.54±1.08	22.31±1.03
	Flooded	15.53±0.31 NS	15.69±0.72 NS	16.00±0.85 NS	16.44±1.24*	17.51±0.74**
Root mass ratio (g·g^−1^)	Non-flooded	0.65±0.02	0.59±0.01	0.63±0.04	0.73±0.03	0.69±0.02
	Flooded	0.64±0.03 NS	0.55±0.02 NS	0.49±0.01*	0.43±0.01**	0.49±0.02**

*, **: significant differences (P<0.05 and P<0.01, respectively) based on a t-test.

### Photosynthesis

**Figure 1:**
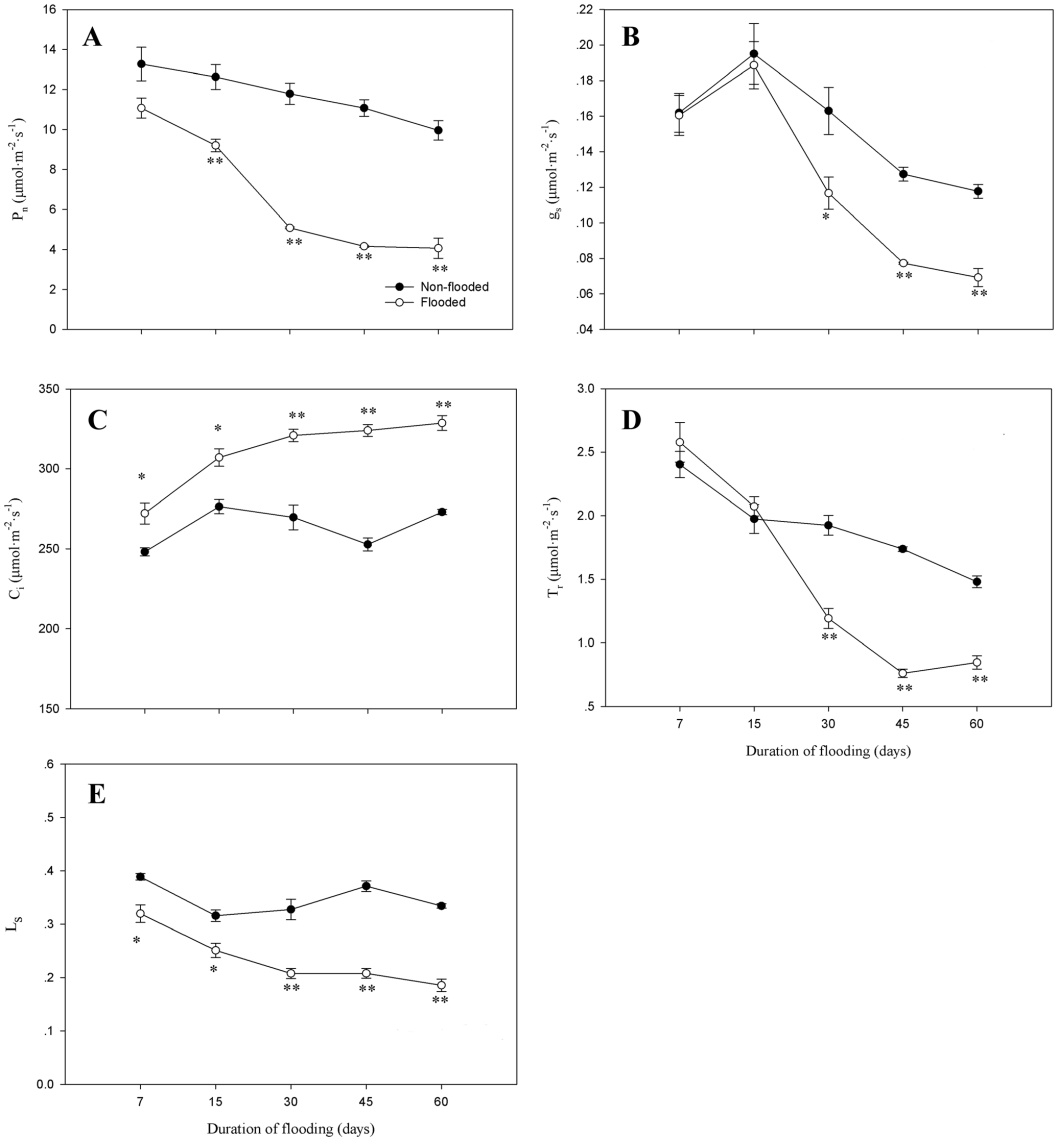
*P*_n_, *g*_s_, *C*_i_, *T*_r_, and *L*_s_ in leaves of *D.chinense* in response to flooding. Means and standard errors based on three replicates are shown. Significant difference induced by flooding is indicated by asterisk: * P<0.05 and ** P<0.01.

Compared to non-flooded seedlings, *P*_n_ significantly decreased after 15 days of flooding (P<0.01), with a 59.2% reduction compared to non-flooded controls by the last day of flooding (Fig. 1A). In contrast, *g*_s_ and *T*_r_ did not significantly decrease until 30 days of flooding (P<0.05); values remained significantly low during the remainder of the flooding period (P<0.01; Fig. 1B, D). *C*_i_ was significantly (P<0.05) higher in flooded seedlings than in non-flooded seedlings beginning at 7 days of flooding, whereas *L*_s_ gradually decreased with a prolonged duration of flooding (Fig. 1 C, E).

**Figure 2:**
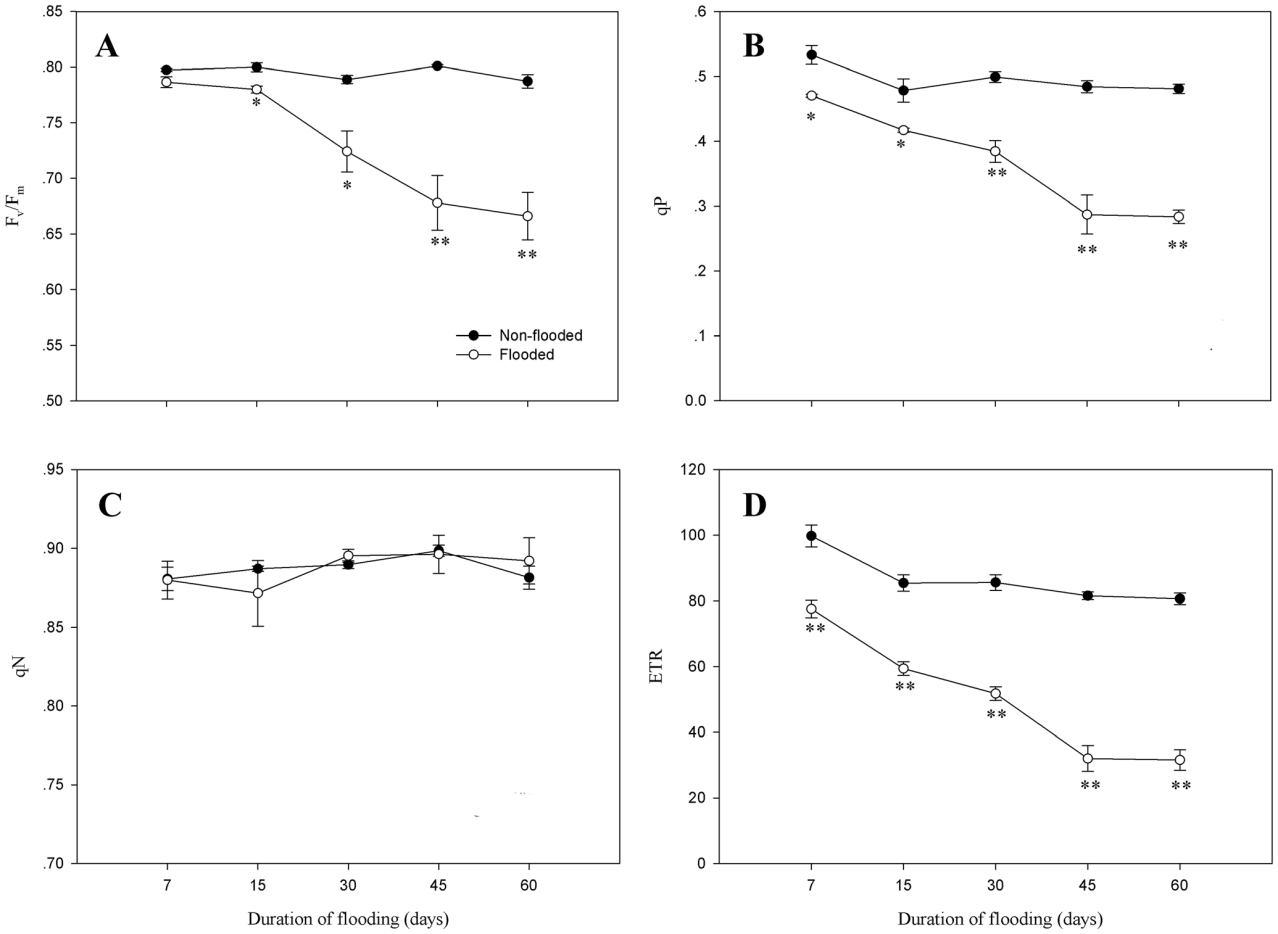
The effect of flooding on Fv/Fm, qP, qN, and ETR in leaves of *D.chinense*. Means and standard errors based on three replicates are shown. Significant difference induced by flooding is indicated by asterisk: * P<0.05 and ** P<0.01.

Fv/Fm was not affected during 7 days of flooding, but beginning at 15 days of flooding, values decreased significantly (P<0.05), with up to a 15.4% reduction by day 60 (P<0.01; Fig. 2A). qP and ETR significantly decreased (P<0.05) during the flooding period, with values 41.0% and 60.8% lower, respectively, than non-flooded seedlings (Fig. 2B, D). qN did not significantly vary between flooded and non-flooded seedlings during the flooding period (Fig. 2C).

### Carbohydrate partitioning

Soluble sugar concentration was significantly greater in leaves (P<0.05) and roots (P<0.01) of flooded seedlings than in non-flooded seedlings throughout 60 days of flooding (Table 3). The leaf soluble sugar concentration in flooded seedlings was 120.2%, and the root soluble sugar concentration was 117.8% of the concentration in non-flooded seedlings. The soluble sugar concentration in stems did not significantly vary between flooded and non-flooded seedlings on day 60.

**Table 3:** Soluble sugar and starch content (means ± S.E., n = 3) of *D.chinense* seedlings under non-flooded and flooded conditions.

Organ	Soluble sugar (mg•g^−1^ dry weight)		Starch (mg•g^−1^ dry weight)	
	Non-flooded	Flooded	Non-flooded	Flooded
Leaf	22.20±1.323	26.69±0.387*	38.39±3.358	54.73±3.924*
Stem	17.17±0.365	16.42±0.150 NS	64.46±1.688	67.68±2.429 NS
Root	16.36±0.286	19.28±0.224**	88.03±5.432	44.96±3.800**

*,**: significant differences (P<0.05 and P<0.01, respectively) based on a t-test.

Flooding significantly (P<0.05) increased the concentration of starch in leaves after 60 days of flooding, with values 42.6% higher than in non-flooded seedlings (Table 3). The concentration of starch in roots was lower in flooded seedlings (P<0.01) and decreased by 48.9% compared to non-flooded seedlings.

### Nutrient concentration

**Figure 3:**
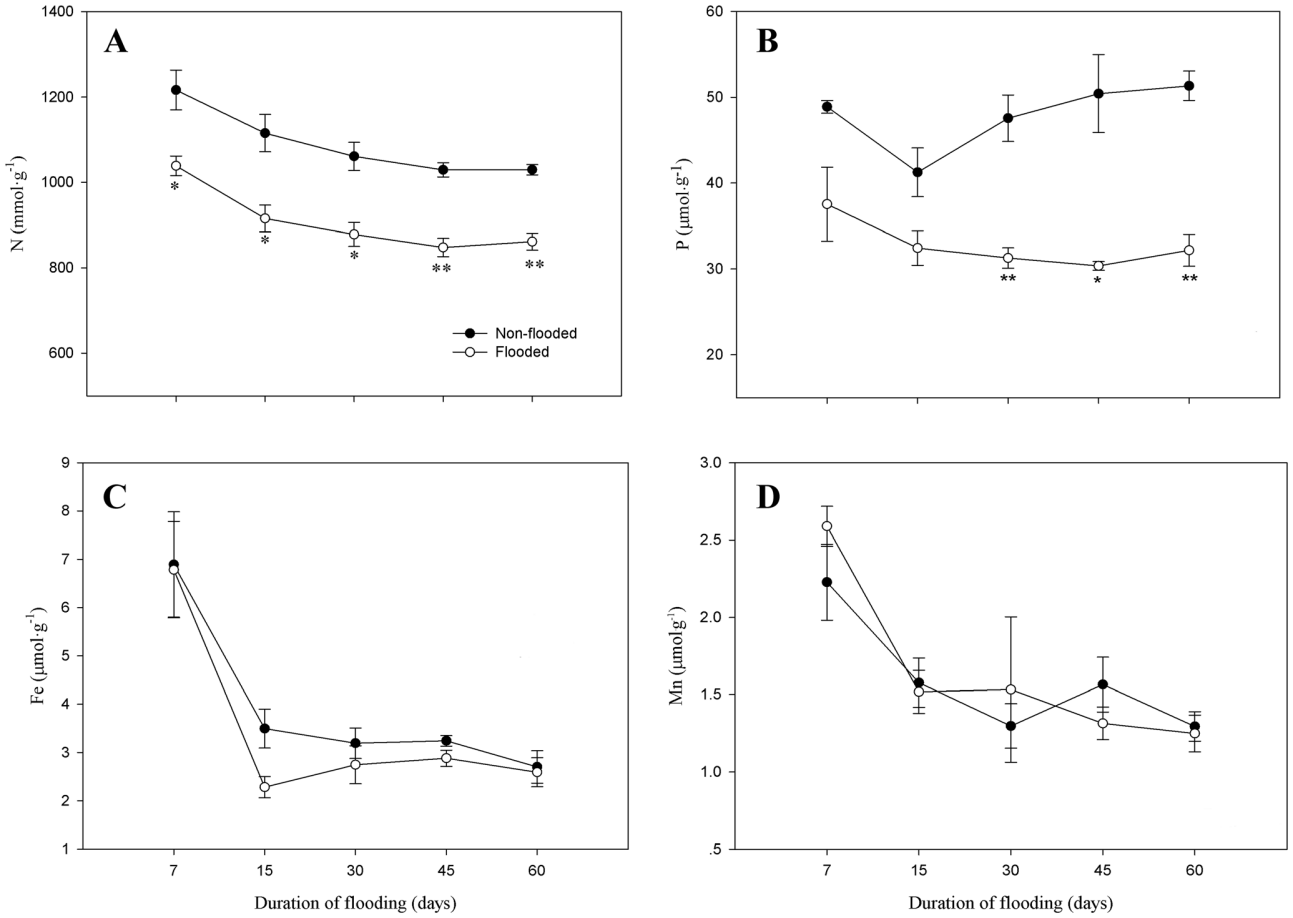
Effect of flooding on nutrient concentrations in leaves of *D.chinense* seedlings. Means and standard errors based on three replicates are shown. Significant difference induced by flooding is indicated by asterisk: * P<0.05 and ** P<0.01.

Concentrations of N and P in leaves significantly varied (P<0.05) in flooded and non-flooded seedlings, whereas Fe and Mn did not (Fig. 3A–D). N in leaves significantly decreased (P<0.05) during the flooding period, with values 16.8% lower than in non-flooded seedlings. On days 7 and 15, the concentrations of P in leaves of flooded seedlings did not markedly differ compared to non-flooded controls. However, beginning at day 30, values decreased significantly (P<0.05), with up to a 48.7% reduction at day 60.

**Figure 4:**
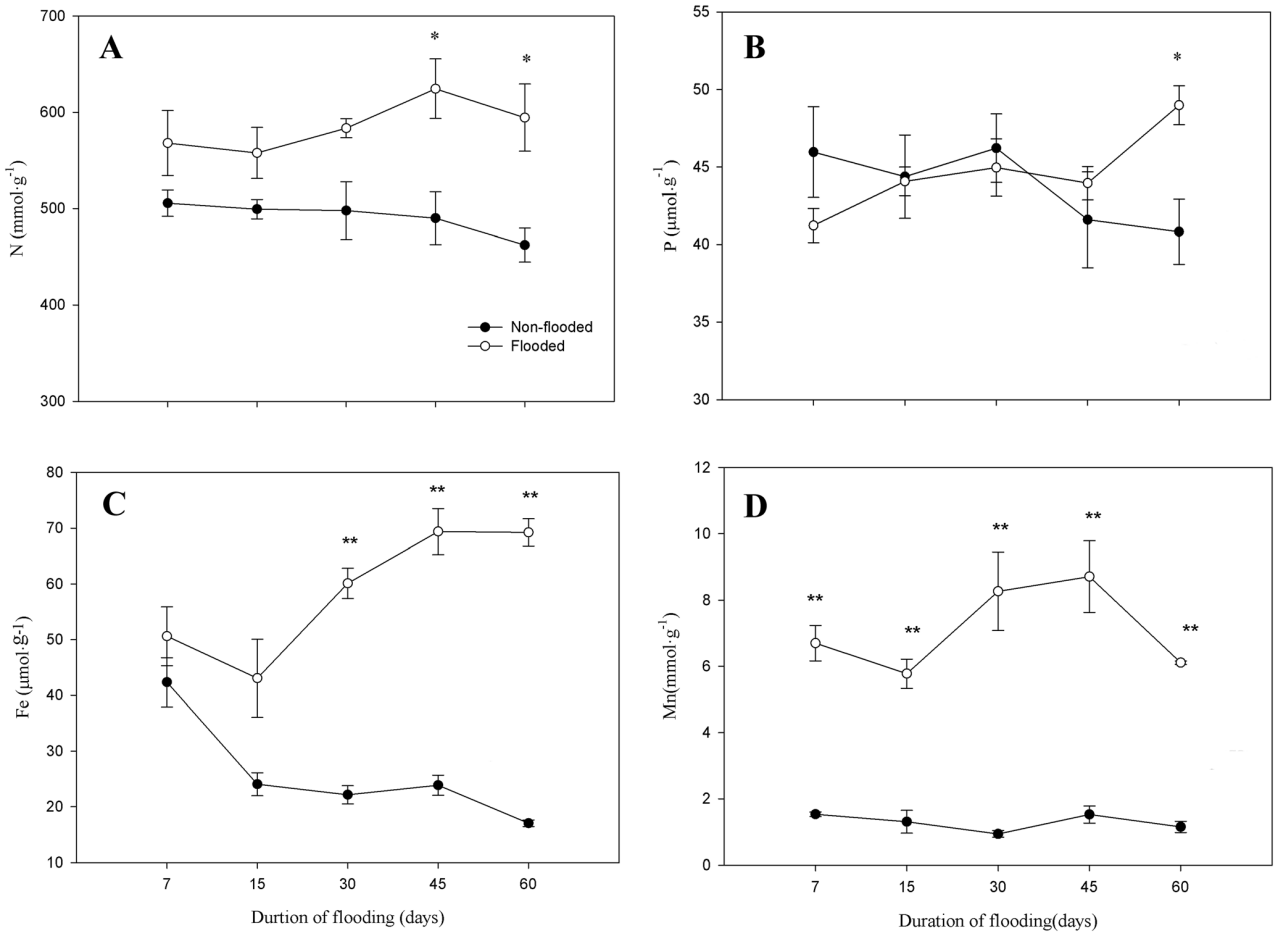
Effect of flooding on nutrient concentrations in roots of *D.chinense* seedlings. Means and standard errors based on three replicates are shown. Significant difference induced by flooding is indicated by asterisk: * P<0.05 and ** P<0.01.

The concentrations of all nutrients in roots of flooded seedlings increased during the process of flooding compared to non-flooded seedling (Fig. 4A–D). N in roots was not affected by 30 days of flooding, but beginning at day 45, values increased significantly (P<0.05). P concentrations in flooded seedlings were significantly higher than in non-flooded seedlings until day 60. The concentrations of Fe in roots were significantly higher (P<0.01) than in non-flooded roots 30 days after flooding. Mn was significantly greater (P<0.01) in roots of flooded seedlings than in non-flooded seedlings throughout the 60 days of flooding.

## Discussion

Our results highlight the high flooding tolerance of *D. chinense*, as indicated by the 100% survival rate and only a slight chlorosis of the older leaves. Similar results have been reported by Peng et al. [27], who found that the survival rate of *D. chinense* seedlings was 100% when faced with 30-days summer flooding, with no symptoms of severe injury. Furthermore, several studies on the effects of flooding in woody plants have demonstrated that adjustments in morphology (such as plant height, stem diameter, adventitious roots, and hypertrophic lenticels) contribute to flood tolerance [28], [29]. In the present study, plant height and stem diameter did not obviously increase, but hypertrophic lenticels and abundant adventitious roots were produced on the submerged portions of stems, which can improve oxygen diffusion from aerated parts to the root system [3]. These changes indicate that *D. chinense* achieves flood tolerance by producing hypertrophic lenticels and adventitious roots rather than by increasing plant height and stem diameter during winter flooding.

Decreases in whole plant biomass and root biomass have been reported as characteristics of some flood-tolerant species such as *Erythrina speciosa* Andrews [30], *Annona glabra* L. [31], and *Tabebuia avellanedae* (Martius ex A.P. de Candolle) [32]. These characteristics were also observed in the present study of *D. chinense*. Slow metabolic activity under hypoxia may be one factor causing these reductions, as the lack of oxygen blocks mitochondrial electron transport, oxidation of NADH+H^+^, and ATP synthesis [33]. However, this loss of root biomass may also be due to the replacement of old tissues by new tissue formation, such as adventitious roots, aerenchymas, and new leaves, which can be more adaptive to the new anaerobic conditions [34].

Decreases in photosynthesis under flooding conditions have been demonstrated in many woody species [35]. Several studies have shown that *P*_n_ was appreciably reduced within hours to a few days after flooding was initiated, but with a prolonged duration of flooding, flood-tolerant plants will produce certain adaptations to flooding and *P*_n_ gradually returns to normal levels or achieves a relatively stable state [36], [37], [38]. In the present experiment, *P*_n_ decreased significantly in early flooding but gradually reached a relatively stable level as the flooding duration continued to increase, maintaining approximately 40% the value observed in non-flooded seedlings. The maintenance of a stable *P*_n_ indicates that *D. chinense* possesses a strong adaptability to flooding, which may be one factor enabling its survival and growth in flooded areas.

*g*_s_ also decreased with flooding in the present experiment, and no stomatal reopening during flooding was observed, even though hypertrophy occurred at submerged portions of stems. Mielke et al. [33] reported similar results for the flood-tolerant *Genipa americana* (Huito). The change in *g*_s_ significantly affected the plant water status. Flooding affects root metabolism and reduces water uptake capability, and a low *g*_s_ can prevent excessive water loss by transpiration to maintain a positive water balance [35], [39]. The decrease of *T*_r_ and the reduction of *g*_s_ in the present experiment also support this idea. Thus, partial stomatal closure should be considered a strategy that contributes to flood tolerance under flooding conditions [33].

The primary causes of decreases in photosynthesis under flooding conditions mainly involve two pathways. First, reductions in photosynthesis may be related to the closure of stomata, which limits the ability of the plant to capture CO_2_, thus reducing photosynthetic enzyme substrate and ultimately lowering the net photosynthetic rate [40]. Second, photosynthesis may also decrease because of non-stomatal limitations, such as decreases in the 1,5-ribulose bisphosphate carboxylase/oxygenase enzyme content and activity [31], [41]; the inhibition of photosynthetic electron transport, photosynthetic phosphorylation, and 1,5-ribulose bisphosphate regeneration [5], [42]; the burden of feeding regulation caused by assimilate transport [33]; the metabolic disorders of active oxygen [5]; and increases in ethylene and other endogenous hormones [42]. All of these processes would affect photosynthesis. Farquhar and Sharkey [40] hypothesized that a decline in the photosynthesis rate would be mainly attributable to stomatal limitation only if both *P*_n_ and *C*_i_ decline with an increase in *L*_s_. In contrast, if *C*_i_ increases in response to changes in *P*_n_ with the decline of *L*_s_, the decline in the photosynthesis rate should be mainly attributed to non-stomatal limitation. Both *P*_n_ and *L*_s_ in *D. chinense* declined with the prolonged duration of flooding, whereas *C*_i_ gradually increased with an increased duration of flooding. Thus, the main factor causing the decrease of the net photosynthetic rate was likely non-stomatal limitation. Studies of riparian plants such as *G. americana* [33], *Salix variegata* Franch. [43], *Pinus elliottii* Engelm. [44], and *Cyperus rotundus* L. (Rhizoma Cyperi) [17] found similar results.

The Chl fluorescence parameter is a very sensitive indicator of photodamage under stress conditions [45]. Fv/Fm is thought to measure the PSII potential photochemical activity. A decrease in Fv/Fm can indicate photoinhibitory impairment from flooding stress. The maintenance of a stable Fv/Fm indicates flooding does not affect the PSII photochemical reaction [33], [46]. In the present experiment, Fv/Fm in flooded seedlings decreased with a prolonged duration of flooding, indicating that the PSII photochemical activity of *D. chinense* after flooding was damaged. qP reflects the energy captured by the PSII reaction center used for photochemical electron transfer, as well as the photosynthetic efficiency and degree of utilization of energy, to a certain extent [47]. qP gradually decreased in flooded seedlings during the flooding period, which coincided with changes in ETR. This finding demonstrates that the photosynthetic electron transport rate and the energy conversion efficiency of *D. chinense* were affected by flooding. qN reflects the energy used in thermal energy dissipation. A decrease in Fv/Fm, qP, and ETR accompanied by an increase in qN may reflect increased photoprotection through the xanthophyll-II cycle rather than photodamage [48]. In the present study, the decreases in Fv/Fm, qP, and ETR and the absence of a compensatory increase in qN with decreases in the net photosynthetic rate after flooding corroborate the result that the drop in net carbon assimilation also occurred via non-stomatal limitation. Based on research of four flood-tolerant tree species, Rengifo et al. [49] also hypothesized that the decrease in the net photosynthetic rate during flooding was associated with a decreases in Fv/Fm and the maximum quantum yield of PSII, as well as the absence of a compensatory increase in qN.

The reduction in photosynthesis accompanied by increased concentrations of starch in leaves and decreased concentrations of starch in roots has also been demonstrated in many flood-tolerant species such as *Lepidium latifolium* L. [42] and *Fraxinus pennsylvanica* Marshall [14]. The inhibition of photosynthate delivery from leaves to roots may result in starch accumulation in leaves of flooded plants and in turn inhibit photosynthesis [50]. In our experiment, flooding increased the starch concentrations of *D. chinense* in leaves but decreased concentrations in roots, suggesting that starch accumulation in *D. chinense* leaves might be a non-stomatal cause of the decrease in photosynthesis. Furthermore, previous studies have shown that the survival of plants after flooding depends on the level of carbohydrate remaining in roots [51]. This response, which was also highlighted by Greenway and Setter [52], is one important flood-tolerance strategy of flood-tolerant plants. The concentration of starch in *D. chinense* after 60 days flooding was maintained at 48.9% of the levels of non-flooded seedlings, implying that maintaining a certain amount of starch reserves might be an adaptation of *D. chinense*, providing energy needed to survive under long-term flooding stress and allowing for a more rapid recovery after a return to aerobic conditions.

Flooding, drought, salinity, and low temperature generally increase the concentrations of soluble sugar [53]. In our experiment, flooding increased the soluble sugar concentrations of *D. chinense* in both leaves and roots. The accumulation of soluble sugar in roots of *D. chinense* suggests that the availability of soluble sugar is not limiting to the metabolic activity in roots. Similar results have been reported by Chen et al. [42], who hypothesized that the increase in soluble sugar might be a product of ethylene-induced enzymatic breakdown in root cellulose to produce aerenchyma.

The concentrations of N and P in leaves play an important role in photosynthesis; therefore, deficiencies in N and P likely contribute to decreases in photosynthetic efficiency. In this study, the concentrations of N and P in leaves were lower in flooded seedlings than in non-flooded seedlings, contributing to the decrease in photosynthesis under flooding conditions. With a prolonged duration of flooding, the concentrations of N and P in leaves did not always decline but gradually stabilized, consistent with changes in the net photosynthetic rate; therefore, the relatively stable concentrations of N and P in leaves may be an important factor ensuring a stable net photosynthetic rate post-flooding. We also found greater concentrations of N and P in the roots of flooded plants. Flooding may inhibit the transport of N and P from roots to leaves, causing N and P enrichment in roots. In addition, a strategy of resource allocation by which N and P enrichment occurs in roots may be advantageous for the post-flooding recovery of stressed *D. chinense*. This resource allocation differs from flood-intolerant species, in which flooding causes significant declines in the uptake of N and P [54].

Fe and Mn are also important components in the functional units of photosynthesis [6]. Neither Fe nor Mn in leaves was significantly affected by flooding in the present study. Fe and Mn concentrations do not appear to be limiting for photosynthetic functioning. In flooded soil, soil redox potential decreases and Fe^2+^ and Mn^2+^ concentrations increase, and soluble Fe^2+^ and Mn^2+^ can lead to excessive uptake during periods of flooding [42]. In our experiment, Fe and Mn concentrations in roots of flooded seedlings were significantly higher than in non-flooded seedlings, which supports this conclusion. However, excessive uptake of Fe^2+^ and Mn^2+^ will produce toxic effects on root tissue [55], [56]. In this study, the uptake of Fe and Mn gradually stabilized or decreased at the end of flooding period, which may be a positive response to the approach toward levels toxic to *D. chinense*.

To summarize, we conclude that the ability of *D. chinense* to acclimate to a long period of flooding depends on a combination of morphological and physiological responses. Morphological adjustments such as the formation of adventitious roots and stem lenticels increase the rate of oxygen diffusion from aerated parts to the root, which is essential for maintaining survival and growth. In addition, the flooding tolerance of *D. chinense* may be closely related to the adjustment capacity, including the maintenance of a stable photosynthetic rate, Fv/Fm, qP, ETR, and nutrient content (N and P) in leaves and maintaining a carbohydrate reserve in roots with a prolonged duration of flooding. To the best of our knowledge, this is the first report on the morphological and physiological responses of *D. chinense* to off-season flooding. This study identifies a series of acclimations to flooding in this flood-tolerant species; however, whether these acclimations can occur or be maintained under longer flooding periods must be examined further.
